# 

**Published:** 1884-08

**Authors:** 


					﻿AUGUST, 1884.
THIRTY-FIRST YEAR.
HALL’S
JOURNAL
OF
HEALTH
Guaranteed Circulation 200,000 Copies in 12 liontliSi
E. H. GIBBS, A.M., M.D., Editor.,
No. 21 CLINTON PLACE EIGHTH STREET NEW YORK.
TERMS: $r a Year.
Single nnmljer, 10 cts.
				

